# Motion compensated reconstruction for myocardial perfusion MRI

**DOI:** 10.1186/1532-429X-14-S1-M11

**Published:** 2012-02-01

**Authors:** Sajan Goud Lingala, Edward DiBella, Christophe Chefd'hotel, Mariappan Nadar, Mathews Jacob

**Affiliations:** 1 Biomedical Engineering, The University of Iowa, Iowa City, IA, USA; 2 Radiology, University of Utah, Salt Lake City, UT, USA; 3 Siemens Corporate Research, Princeton, NJ, USA; 4 Electrical and Computer Engineering, The University of Iowa, Iowa City, IA, USA

## Background

Classical acceleration schemes for myocardial perfusion MRI like view sharing, k-t BLAST [Tsao et al. ‘03] are sensitive to motion artifacts which could arise in data with inconsistent gating and/or breathing motion. A natural approach to be robust to this is to estimate the motion and compensate for it during the recovery; to this end we proposed a novel joint motion estimation and reconstruction scheme in [Lingala et al. ‘11]. One goal of this work is to further validate it by recovering such data at considerable accelerations (R). In a 2nd goal, we apply it to recover images acquired with an ungated protocol [DiBella et al. ‘11]. This obtains data at a rapid rate without any gating to provide robustness to beat-beat variability. It also offers a high temporal resolution (~50ms), which ensures maximal information is obtained during the brief first pass. This resolution also means the cardiac motion is captured, akin to cine imaging. Here, we aim to benefit the reconstructions by compensating for this motion. We show its utility by comparisons with standard gridding reconstruction (GR) and sliding window (SW) algorithm.

## Methods

**Figure 1:**
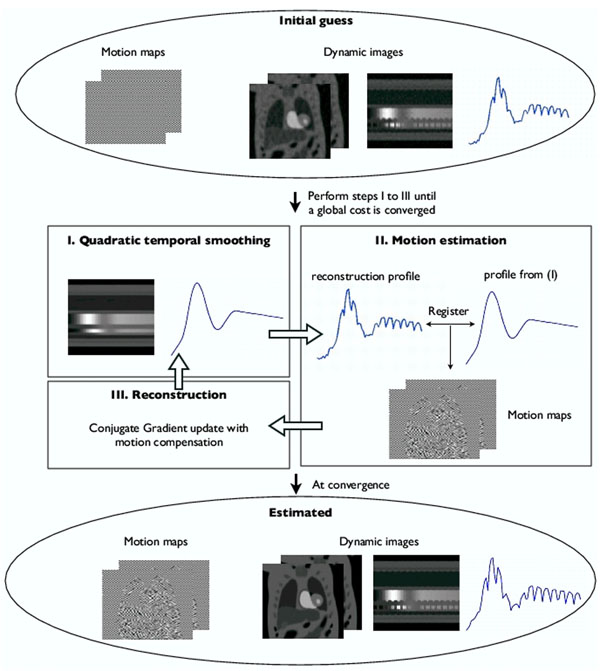
The motion compensated recovery algorithm*: The proposed scheme jointly estimates the images and motion by minimizing a cost function which penalizes the roughness of the pixel time series subject to data consistency. In an iterative mode, it first estimates a reference dynamic scene that is free of motion by using quadratic temporal smoothing. Using this, the motion is estimated with a non-rigid coarse-fine deformation model based on maximizing the normalized cross correlation. With the motion estimates, a spline based interpolant was used to warp the images in the CG step.*Here, we show the flow chart using a physiological PINCAT [Shariff et al. 07] phantom.

**Figure 2:**
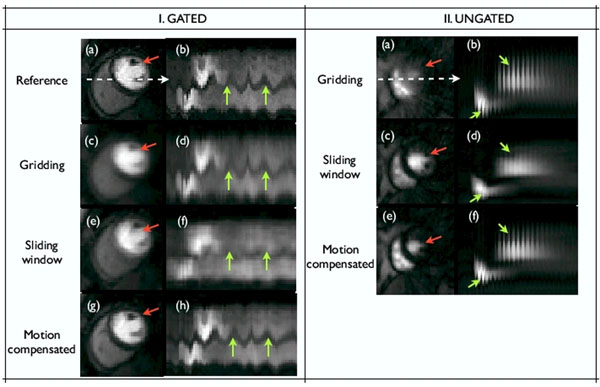
Recovery of perfusion data with (I) inconsistent gating and shallow breathing and (II) ungated data. Few spatial frames and the image time profiles are shown. In (I), the first row shows the fully sampled data. The motion due to breathing and improper gating are shown by the green arrows. In (II), we consider applying the reconstructions to a single slice; the motion here is due to the cardiac dynamics. Here, we note that the proposed scheme robustly recovers the data in the presence of motion, while sliding window and the gridding reconstructions respectively show temporal blur and streak artifacts. Specifically, for the ungated data, we note that if the motion is not compensated, it could lead to misinterpreting the cardiac phase at a particular time frame. For instance in (II a), it is clear that the systolic phase is being imaged, while sliding window in (II b) depicts this as a phase which looks like diastolic. The proposed scheme recovers the appropriate phase in (II c).

The proposed method exploits the temporal smoothness of the contrast variations, and assumes the changes due to motion are less smooth. The motion and the images are recovered jointly in an iterative framework, where the roughness of the motion compensated pixel time profiles are penalized subject to data consistency. It iterates between steps of quadratic temporal smoothing, motion estimation and conjugate gradient update of the reconstructions (fig 1). To validate, a saturation recovery FLASH sequence (TR/TE=2.5/1ms) was used with gating to acquire data on a Cartesian grid (PE x FE encodes: 90x190, temporal resolution: 1 beat). A subject with some heart rate variability was imaged during shallow breathing, and some integer shifts were added to amplify the motion (see fig 2). Resampling experiments on a radial grid were done at R~4. In a 2nd protocol, radial k space data with no gating was acquired (TR/TE=2.2/1.2 ms, 2.3x2.3mm pixel, 20 interleaved rays/frame with rotations across frames). 5 slices were acquired after each saturation pulse, during a breath hold. This gave a total of 280 images/slice, where each image was acquired in 44 ms, and repeated every ~270 ms (fig 2).

## Results

From fig 2, the images recovered with the proposed scheme were robust to motion blur and streaking artifacts seen respectively with the SW and the GR methods. Apart from its use in the recovery, the motion estimates here could be used for subsequent post-processing. For instance, to improve the temporal resolution and/or the SNR of the ungated images or to implement self-gating.

## Conclusions

A novel motion compensation scheme for recovering perfusion images with significant motion content was demonstrated. The experiments with both inconsistent gated, breathing and ungated data show promise; further studies on multiple sets are required to thoroughly evaluate the method.

## Funding

NSF AWARD CCF-0844812 and in part by NIH R01EB006155.

